# The Influence of Radio-Frequency Transmit Field Inhomogeneities on the Accuracy of G-ratio Weighted Imaging

**DOI:** 10.3389/fnins.2021.674719

**Published:** 2021-07-05

**Authors:** Tim M. Emmenegger, Gergely David, Mohammad Ashtarayeh, Francisco J. Fritz, Isabel Ellerbrock, Gunther Helms, Evelyne Balteau, Patrick Freund, Siawoosh Mohammadi

**Affiliations:** ^1^Spinal Cord Injury Center Balgrist, University Hospital Zurich, University of Zurich, Zurich, Switzerland; ^2^Department of Systems Neuroscience, University Medical Center Hamburg-Eppendorf, Hamburg, Germany; ^3^Department of Clinical Neuroscience, Karolinska Institutet, Stockholm, Sweden; ^4^Medical Radiation Physics, Clinical Sciences Lund (IKVL), Lund University, Lund, Sweden; ^5^GIGA Institute, University of Liège, Liège, Belgium; ^6^Department of Neurophysics, Max Planck Institute for Human Cognitive and Brain Sciences, Leipzig, Germany; ^7^Wellcome Trust Centre for Neuroimaging, University College London, London, United Kingdom

**Keywords:** myelin volume fraction, axon volume fraction, radio-frequency transmit field inhomogeneities, B_1_+ correction, multi-parameter mapping, diffusion MRI, magnetization transfer saturation, MR g-ratio

## Abstract

G-ratio weighted imaging is a non-invasive, *in-vivo* MRI-based technique that aims at estimating an aggregated measure of relative myelination of axons across the entire brain white matter. The MR g-ratio and its constituents (axonal and myelin volume fraction) are more specific to the tissue microstructure than conventional MRI metrics targeting either the myelin or axonal compartment. To calculate the MR g-ratio, an MRI-based myelin-mapping technique is combined with an axon-sensitive MR technique (such as diffusion MRI). Correction for radio-frequency transmit (B1+) field inhomogeneities is crucial for myelin mapping techniques such as magnetization transfer saturation. Here we assessed the effect of B1+ correction on g-ratio weighted imaging. To this end, the B1+ field was measured and the B1+ corrected MR g-ratio was used as the reference in a Bland-Altman analysis. We found a substantial bias (≈-89%) and error (≈37%) relative to the dynamic range of g-ratio values in the white matter if the B1+ correction was not applied. Moreover, we tested the efficiency of a data-driven B1+ correction approach that was applied retrospectively without additional reference measurements. We found that it reduced the bias and error in the MR g-ratio by a factor of three. The data-driven correction is readily available in the open-source hMRI toolbox (www.hmri.info) which is embedded in the statistical parameter mapping (SPM) framework.

## Introduction

The g-ratio [i.e., the ratio between the inner (r) and outer (R) radius of an axon with myelin sheath (g-ratio = r/R)] of a given axon quantifies the degree of relative myelination, ranging between 0 (no axon) and 1 (no myelin). The g-ratio captures both axonal and myelin damage by incorporating axonal and myelin volumes in one metric, making it potentially more specific to tissue integrity than focusing on one of these aspects only. For example, in multiple sclerosis, the g-ratio increases if the underlying disease mechanism is solely driven by demyelination (Yu et al., 2019), but is expected to remain unaffected if demyelination is accompanied by axonal degeneration. To differentiate such processes and understand their functional implications, neuroscience and clinical research would greatly benefit from *in-vivo* whole-brain measurements of MR g-ratio. Until recently, the g-ratio was measurable only by means of histology (Hildebrand and Hahn, 1978), which restricted the analyses to a small number of axons and a limited number of small brain regions or pathways. Stikov et al. (2011, 2015) introduced a methodology for an MRI-based whole-brain “aggregate” g-ratio mapping, to which we refer as “MR g-ratio” or “g-ratio weighted imaging.” In g-ratio weighted imaging, the MR g-ratio is computed on a voxel-by-voxel basis from the axonal (AVF) and myelin volume fraction (MVF) maps and reflects a weighted mean of g-ratio values within the voxel (West et al., 2016). Therefore, g-ratio weighted imaging requires the acquisition of separate sets of images that are sensitive to *AVF* and MVF, respectively (Campbell et al., 2018; Mohammadi and Callaghan, 2020). To generate MVF and AVF from the measured MR parameters, a calibration step is required that converts the measured MR-visible water signals into the respective volume fractions (Mohammadi and Callaghan, 2020).

Magnetization transfer saturation (MT_sat_) has often been used as proxy for MVF (Mohammadi et al., 2015) as it is minimally affected by the longitudinal relaxation time (Helms et al., 2008) and is expected to show high correlation with macromolecular content (Sereno et al., 2013; Callaghan et al., 2015a; Campbell et al., 2018), making it a sensitive metric of MVF. One common approach to estimate AVF complements the parameters from neurite orientation and dispersion density imaging (NODDI Zhang et al., 2012) with a MVF-proxy, e.g., MT_sat_ (Ellerbrock and Mohammadi, 2018; Kamagata et al., 2019), to correct for the missing myelin water signal in diffusion MRI measurements (Stikov et al., 2015). Maps of MT_sat_ can be obtained, among others, from the multi-parameter mapping (MPM) protocol (Weiskopf et al., 2013) in combination with the hMRI toolbox^1^ (Callaghan et al., 2019; Tabelow et al., 2019).

Although the MT_sat_ measure is largely insensitive to transmit field (B_1_+) inhomogeneities (Helms et al., 2008), it still shows a residual dependence which introduces a bias and/or error in the MT_sat_ maps that can propagate into the MR g-ratio and lead to systematic bias. Such B_1_+ inhomogeneities can be corrected based on an independently acquired B_1_+ field map measurement (Helms, 2015; Helms et al., 2021). Residual B_1_+ inhomogeneity effects on MT_sat_ have been shown to be not negligible when the B_1_+ correction was omitted (Helms, 2015; Helms et al., 2021). However, the impact of B_1_+ correction on MR g-ratio estimates is unknown. Additionally, it is unclear whether these residual B_1_+ inhomogeneity in MT_sat_ and the MR g-ratio can retrospectively be corrected using a data-driven B_1_+ field inhomogeneities estimation approach such as the “unified segmentation based correction of R1 maps for B_1_+ inhomogeneities” (UNICORT, (Weiskopf et al., 2011)).

In this study, we investigate the effect of B_1_+ inhomogeneities on MR g-ratio maps when omitting the B_1_+ correction. As a reference, we use the B_1_+ corrected MR g-ratio from a dataset of healthy controls. We compare the reference MR g-ratio values against (i) values obtained without B_1_+ correction and (ii) values obtained with B_1_+ correction using the data-driven UNICORT approach.

## Materials and Methods

### Subjects

This study included 25 healthy control subjects (12 females, age (mean ± standard deviation) of 25.4 ± 2.4 years). They were recruited at the University Medical Centre Hamburg-Eppendorf and screened for neurological or psychiatric illness. The study was in agreement with the Declaration of Helsinki and was approved by the local ethics committee (Ärztekammer Hamburg #PV5141).

### Data Acquisition

Each subject was scanned twice within 1 week in a whole-body 3T Tim TRIO MR scanner (Siemens Healthcare, Erlangen, Germany) using the body RF-coil for transmission and a 32-channel radiofrequency (RF) head coil for signal reception, respectively. The MR acquisition on both scan days included a multi-parameter mapping (MPM) (Weiskopf et al., 2013; Callaghan et al., 2015b) and a diffusion-weighted imaging (DWI) protocol. The MPM protocol consists of three differently weighted 3D-multi-echo spoiled gradient echo sequences (Siemens FLASH). The echo train length and flip angle for the proton density (PD) weighted, T1-weighted, and magnetization transfer (MT) weighted sequences were 8/6, 8/21, and 6/6°, respectively. The MT-weighted sequence had a Gaussian RF pulse (2 kHz off resonance with 4 ms duration and a nominal flip angle of 220°). All other sequence parameters were the same for the three sequences: repetition time (TR) 25 ms, echo spacing, resolution 0.8 mm isotropic; field of view (FoV) 166 × 224 × 256 mm^3^, readout bandwidth 488 Hz/pixel, partially parallel imaging using the GRAPPA algorithm was employed in each phase-encoded direction (anterior-posterior and right-left) with 40 reference lines and a speed up factor of two, total acquisition time: ∼25 min. The B_1_+ field reference map was acquired using the three-dimensional echo-planar imaging (3D EPI) method, including field maps for distortion correction (Lutti et al., 2010).

The DWI sequence was a twice-refocused single-shot spin-echo EPI scheme (Reese et al., 2003), consisting of 12 non-diffusion-weighted images (b_0_ images), equidistantly distributed across the diffusion weighted images. The diffusion-weighted images were acquired at two *b*-values (1000$\frac{s}{mm^{2}}$ and 2000$\frac{s}{mm^{2}}$), sampled along 60 unique diffusion-gradient directions within each shell. The entire protocol was repeated with identical parameters but with reversed phase encoding direction (anterior-posterior) to correct for susceptibility-related image distortions (blip-up, blip-down correction). In total, 264 images were acquired per subject (120 diffusion-weighted images, 12 b_0_ images, each acquired twice). Other acquisition parameters were: 86 slices with no gap, TR = 7.1 s, TE = 122 ms, an isotropic voxel size of (1.6 mm)^3^, FoV = 224 × 224 × 138 mm^3^, 7/8 partial Fourier imaging in phase encoding direction, readout bandwidth. To accelerate the data acquisition, GRAPPA (in-plane acceleration with factor two) and simultaneous multi-slice acquisitions (“multiband,” slice acceleration factor two) (Feinberg et al., 2010; Moeller et al., 2010; Xu et al., 2013) were used as described in Setsompop et al. (2012). The image reconstruction algorithm was provided by the University of Minnesota Centre for Magnetic Resonance Research. The total acquisition time was ∼37 min.

### Data Processing

MT_sat_ maps were generated in the SPM-based hMRI toolbox (Tabelow et al., 2019). Note that the hMRI toolbox also generates additional maps of longitudinal (R_1_) and effective transverse relaxation rates (${\text{R}}_{2}^{⋆}$) and PD. Three MT_sat_ maps were generated: (i) ${\text{MT}}_{\text{sat}}^{\text{NO}}$ maps, without B_1_+ correction; (ii) ${\text{MT}}_{\text{sat}}^{\mathrm{B1}}$ map, using the reference B_1_+ field map for correction (Lutti et al., 2010); and (iii) ${\text{MT}}_{\text{sat}}^{\text{UN}}$ maps, using the data-driven UNICORT approach for B_1_+ estimation (Weiskopf et al., 2011; see Supplementary Figure 2). UNICORT is a probabilistic framework for unified-segmentation based correction of R_1_ maps for B_1_+ inhomogeneities. The framework incorporates a physically informed generative model of smooth B_1_+ inhomogeneities and their multiplicative effect on R_1_ estimates (Weiskopf et al., 2011). Parameters used in UNICORT such as the smoothness and regularization were optimized for R_1_ B_1_+ correction in a 3T scanner (i.e., Tim Trio scanner—Weiskopf et al., 2011).

For B_1_+ correction, we used the following heuristic correction factor as detailed in Helms (2015), and Helms et al. (2021):


(1) $${\text{MT}}_{\text{sat}}^{\text{Corr}}={\text{MT}}_{\text{sat}}^{\text{NO}}\frac{1-C}{1-C{\text{B}}_{1}^{+}},$$

where *C* has been calibrated to be 0.4 for the MT pulse used in this paper. B_1_+ can be either measured (${\text{MT}}_{\text{sat}}^{\text{Corr}}={\text{MT}}_{\text{sat}}^{\mathrm{B1}}$) or estimated with the UNICORT approach (${\text{MT}}_{\text{sat}}^{\text{Corr}}={\text{MT}}_{\text{sat}}^{\text{UN}}$).

The DWI data were processed based on the pipeline described in Ellerbrock and Mohammadi (2018) using the SPM-based ACID toolbox^2^. It included several artifact corrections such as Rician signal bias correction (i.e., denoising) (André et al., 2014), correction for eddy current and motion artifacts (Mohammadi et al., 2010, 2014), and correction for image distortions due to susceptibility artifact using reversed phase encoding (Ruthotto et al., 2012, 2013; Macdonald and Ruthotto, 2018). The corrected images were fitted with the NODDI signal model (Zhang et al., 2012) to estimate the intra-cellular volume fraction (ν_*icvf*_), the isotropic volume fraction (ν_*iso*_), and the orientation dispersion index (ODI) in each voxel.

### Spatial Alignment

#### Co-registration

**FIGURE 1:**
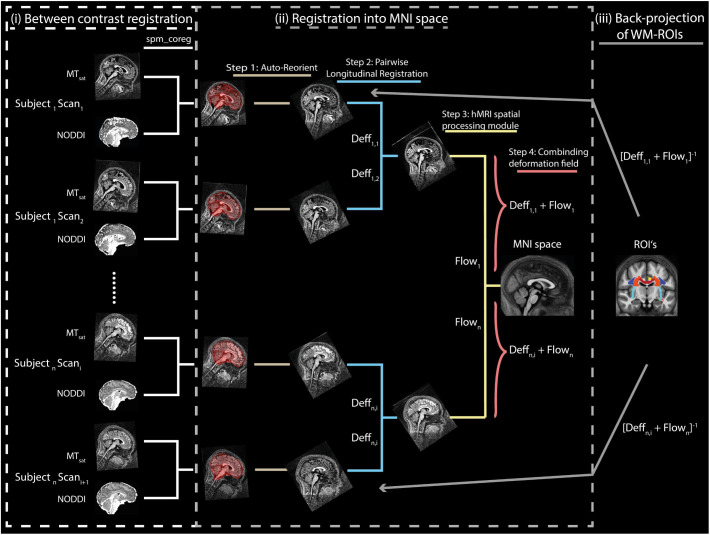
Illustration of the spatial alignment pipeline of the MT_sat_ and NODDI maps. The pipeline consists of (i) co-registration between MT_sat_ and NODDI maps (driven by ODI map), (ii) normalization into MNI space, and (iii) back-projection of ROIs into the native space. Note that each subject consists of two sets of images acquired in separate sessions. In the co-registration step (section “Co-registration”), the white matter (WM) tissue probability map (TPM) of the ODI was co-registered to the WM TMP of the MT_sat_ in each subject and session using rigid-body registration (*spm_coreg* algorithm, SPM12). The resulting transformation was applied to all other NODDI maps as well. In the normalization step (section “Normalization”), MT_sat_ maps were roughly aligned with the T1-weighted MNI template in each subject and session using the *Auto-Reorient* function. The realigned MT_sat_ maps from both sessions were then registered to their mid-point average using the Pairwise Longitudinal Registration (SPM12). In each subject, the mid-point average MT_sat_ map was normalized to the MNI space using the DARTEL-based (Ashburner, 2007) Spatial Processing module. Finally, all deformation fields were converted to a single deformation field and applied on the NODDI maps. In the last step (section “Region of Interest Selection”), the ROIs and the WM masks were back-projected into the native space using the inverse of the combined deformation field.

The voxel-wise arithmetic between the MT_sat_ and ν_icvf_ maps, necessary for MR g-ratio computation, requires an accurate spatial alignment between the two maps (Mohammadi et al., 2015). To this end, we created two white matter (WM) tissue probability maps (TPMs) based on the ODI and ${\text{MT}}_{\text{sat}}^{\mathrm{B1}}$ maps, respectively (Figure 1). To reduce the influence of contrast-specific artifacts (e.g., due to subject motion) on the registration quality, the WM TPM of the ODI map was co-registered to the WM TPM of the ${\text{MT}}_{\text{sat}}^{\mathrm{B1}}$ map using rigid-body registration (*spm_coreg* algorithm, SPM toolbox). The estimated transformation parameters were applied to all other NODDI maps as well. Note that the segmentation quality of the second session was unsatisfactory for two subjects, and the ${\text{R}}_{1}^{{\text{B}}_{1}}$ map (R_1_ with B_1_+ inhomogeneities bias correction using the B_1_+ reference measurements) was used to generate the WM TPM instead. In another subject, the ν_iso_ was segmented instead of the ODI to achieve satisfactory WM segments.

#### Normalization

Spatial normalization was performed in four steps. First, a rough alignment of the ${\text{MT}}_{\text{sat}}^{\mathrm{B1}}$ maps with the T1-weighted MNI template image was achieved using the Auto-Reorient function (hMRI toolbox) and this was applied on the NODDI maps as well. Second, both ${\text{MT}}_{\text{sat}}^{\mathrm{B1}}$ maps of each subject (corresponding to two sessions) were registered to the mid-point average using the Pairwise Longitudinal Registration (SPM12). Hereby, values below zero and above 10 were excluded to improve the registration. Third, the resulting mid-point average image was normalized to the MNI space using the DARTEL-based (Ashburner, 2007) Spatial Processing module (hMRI toolbox). Fourth, a combined deformation field was generated per subject and session, combining the deformation fields from steps 2 and 3.

### Computation of MVF_MR_, AVF_MR_ and g_MR_

In this section, our approach to estimating MVF and AVF from the measured MR parameters is introduced. The MR-based MVF (MVF_MR_) was assumed to be proportional to MT_sat_ without intercept, following (Mohammadi and Callaghan, 2020):


(2) $${\text{MVF}}_{\text{MR}}=\alpha {\text{MT}}_{\text{sat}}$$

The proportionality constant α was estimated from Equation (2) in a region where the histological MVF (MVF_hist_) was known. Due to the lack of own histological data, we used published histological data which contain the frequency distribution of inner-axon radius (*r*) and myelin sheath thickness (m) of 2,400 myelinated fibers in the medullary pyramids of a 71 years old human (see Table 1 in Graf von Keyserlingk and Schramm, 1984). The total volume (TV) of the sample is the sum of the total volume of myelinated axons (TAV_m_), unmyelinated axons (TAV_u_), myelin volume (TMV), and extra-cellular volume (TEV). TAV_m_ was calculated as $\sum_{i=1}^{{\text{N}}_{\text{m}}}\pi {\text{r}}_{i}^{2}$ with *i* indexing the N_m_ myelinated axons only, and TMV was computed as $\sum_{i=1}^{{\text{N}}_{\text{m}}}\pi {\left(r_{i}+{\text{m}}_{i}\right)}^{2}-{\text{TAV}}_{\text{m}}$. TAV_u_, while not reported in Graf von Keyserlingk and Schramm (1984), was found to be approximately 43% of TAV_m_ for multiple mammals (Swadlow et al., 1980; LaMantia and Rakic, 1990; Olivares et al., 2001; Wang et al., 2008; Liewald et al., 2014). Note that the aforementioned papers typically reported the unmyelinated axons as 30% of the total volume of axons, which corresponds to 43% (= $\frac{0.3}{1-0.3}\cdot 100$) of TAV_m_. EVF was estimated to be 25%, according to Lehmenkühler et al. (1993), Nicholson and Hrabìtová (2017), Tønnesen et al. (2018). Finally, MVF was calculated as


(3) $${\text{MVF}}_{\text{hist}}\approx \frac{1}{\text{TV}}\sum_{j=1}^{\text{N}}\pi \left({\left({\text{r}}_{j}+{\text{m}}_{j}\right)}^{2}-{\text{r}}_{j}^{2}\right)$$

**TABLE 1:** Group-averaged mean and standard deviation (SD) of ${\text{g}}_{\text{MR}}^{\mathrm{B1}}$, ${\text{MVF}}_{\text{MR}}^{\mathrm{B1}}$, and ${\text{AVF}}_{\text{MR}}^{\mathrm{B1}}$ in 21 high-SNR ROIs.

Name	Acronym	${\text{g}}_{\mathrm{MR}}^{\mathrm{B1}}$ mean ± SD	${\text{AVF}}_{\mathrm{MR}}^{\mathrm{B1}}$ mean ± SD	${\text{MVF}}_{\mathrm{MR}}^{\mathrm{B1}}$ mean ± SD
Anterior limb of internal capsule right	ACL r	0.688 ± 0.029	0.384 ± 0.052	0.419 ± 0.022
Retrolenticular part of internal capsule left	RIC l	0.665 ± 0.020	0.341 ± 0.025	0.428 ± 0.023
Anterior corona radiata right	ACR r	0.651 ± 0.012	0.321 ± 0.014	0.435 ± 0.014
Anterior corona radiata left	ACR l	0.644 ± 0.015	0.313 ± 0.014	0.440 ± 0.018
Superior corona radiata right	SCR r	0.679 ± 0.014	0.356 ± 0.018	0.413 ± 0.087
Superior corona radiata left	SCR l	0.674 ± 0.013	0.350 ± 0.016	0.419 ± 0.017
Genu of corpus callosum	GCC	0.642 ± 0.020	0.315 ± 0.021	0.445 ± 0.024
Body of corpus callosum	BCC	0.657 ± 0.021	0.328 ± 0.025	0.425 ± 0.020
Posterior corona radiata right	PCR r	0.662 ± 0.019	0.326 ± 0.025	0.416 ± 0.019
Posterior corona radiata left	PCR l	0.667 ± 0.018	0.337 ± 0.023	0.418 ± 0.019
Posterior thalamic radiation right	PTR r	0.643 ± 0.016	0.308 ± 0.017	0.438 ± 0.018
Posterior thalamic radiation left	PTR l	0.645 ± 0.017	0.313 ± 0.016	0.438 ± 0.020
Sagittal stratum left	SAS l	0.645 ± 0.021	0.314 ± 0.020	0.439 ± 0.025
External capsule right	EXC r	0.683 ± 0.020	0.359 ± 0.023	0.410 ± 0.028
External capsule left	EXC l	0.682 ± 0.025	0.357 ± 0.023	0.408 ± 0.034
Cingulum left	CGM l	0.661 ± 0.023	0.330 ± 0.028	0.422 ± 0.029
Fornix/Stria terminalis left	FNX l	0.669 ± 0.027	0.349 ± 0.036	0.426 ± 0.028
Superior longitudinal fasciculus right	SLF r	0.666 ± 0.016	0.334 ± 0.017	0.418 ± 0.022
Superior longitudinal fasciculus left	SLF l	0.668 ± 0.013	0.340 ± 0.015	0.420 ± 0.020
Superior fronto-occipital fasciculus right	SFO r	0.678 ± 0.020	0.361 ± 0.031	0.422 ± 0.020
Superior fronto-occipital fasciculus left	SFO l	0.672 ± 0.021	0.350 ± 0.029	0.424 ± 0.020

with j indexing all N fibers, yielding MVF_hist_ ≈ 0.3623. Plugging this value into Equation (2) (assuming that MVF_MR_ ≈ MVF_hist_) along with the group-average MT_sat_ within the medullary pyramids (see Figure 2 for ROI definition) yielded an α of 0.2496 for ${\text{MT}}_{\text{sat}}^{\mathrm{B1}}$, 0.2414 for ${\text{MT}}_{\text{sat}}^{\text{UN}}$, and 0.2884 for ${\text{MT}}_{\text{sat}}^{\text{NO}}$.

**FIGURE 2:**
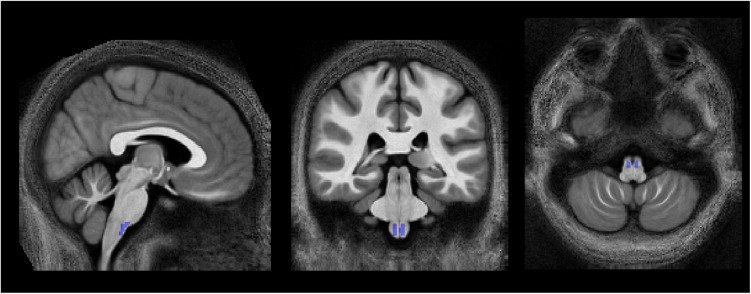
Location of the pyramidal tracts in the medulla oblongata ROI, overlaid on the group-averaged ${\text{MT}}_{\text{sat}}^{\mathrm{B1}}$ map, that was used to determine the calibration constant, converting MT_sat_ into MVF_MR_ (section “Computation of MVF_MR_, AVF_MR_, and g_MR_″). To create this ROI, the corticospinal tract ROI of the JHU-ICBM-DTI-81 atlas, which extends across the pons and medulla pyramids, was modified to cover only the medulla pyramids. Left-right position: X = 82; anterior-posterior position: Y = 77; superior-inferior position, Z = 30.

The MR-based AVF (AVF_MR_ = (1−MVF_MR_)AWF_MR_) was calculated as


(4) $${\text{AVF}}_{\text{MR}}=\left(1-\alpha {\text{MT}}_{\text{sat}}\right)\left(1-\nu_{\text{iso}}\right)\nu_{\text{icvf}}$$

where AWF = (1−ν_iso_)ν_icvf_ is the axonal water fraction estimated from the NODDI parameters (Stikov et al., 2015) and MVF_MR_ = αMT_sat_. The MR g-ratio was then computed according to Stikov et al. (2011, 2015)


(5) $${\text{g}}_{\text{MR}}=\sqrt{1-\frac{{\text{MVF}}_{\text{MR}}}{{\text{MVF}}_{\text{MR}}+{\text{AVF}}_{\text{MR}}}}$$

Note that three versions of MT_sat_, AVF_MR_, and g_MR_ were generated according to notation in section “Data Processing”: (i) ${\text{MVF}}_{\text{MR}}^{\text{NO}}$, ${\text{AVF}}_{\text{MR}}^{\text{NO}}$, ${\text{g}}_{\text{MR}}^{\text{NO}}$ for no correction, (ii) ${\text{MVF}}_{\text{MR}}^{\mathrm{B1}}$, ${\text{AVF}}_{\text{MR}}^{\mathrm{B1}}$, and ${\text{g}}_{\text{MR}}^{\mathrm{B1}}$ for B_1_+ reference measurement, and (iii) ${\text{MVF}}_{\text{MR}}^{\text{UN}}$, ${\text{AVF}}_{\text{MR}}^{\text{UN}}$, and ${\text{g}}_{\text{MR}}^{\text{UN}}$ for UNICORT B_1_+ correction.

### Definition of White Matter Masks

As g_MR_ and its constituents (MVF_MR_, AVF_MR_) are defined only in the WM, we restricted the analysis to the WM by creating binary WM masks (Mohammadi and Callaghan, 2020). WM tissue probability maps (WM-TPM) were created for each subject by segmenting AWF and ${\text{MT}}_{\text{sat}}^{\mathrm{B1}}$ using the hMRI toolbox, and taking their intersection according to Mohammadi and Callaghan (2020). In two subjects, the ${\text{MT}}_{\text{sat}}^{\mathrm{B1}}$ segmentation was of insufficient quality for segmentation and was replaced by the ${\text{R}}_{1}^{{\text{B}}_{1}}$ map. A group-specific binary WM mask (WM_*group*_) was generated by averaging all individual WM-TPMs in the MNI space and thresholding it at 0.95.

**FIGURE 3:**
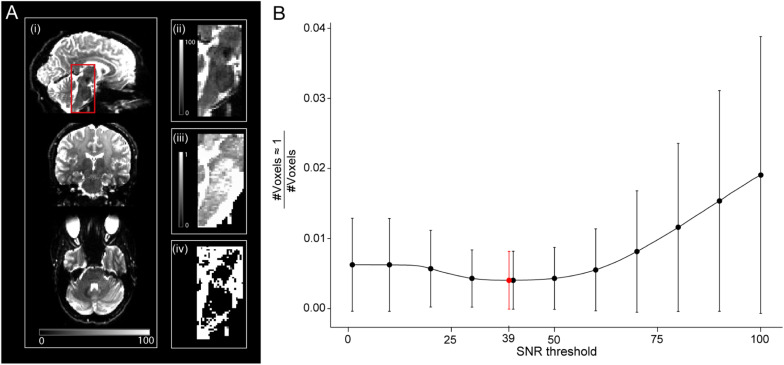
Relationship between signal-to-noise ratio (SNR) and unrealistically high ν_icvf_ values—here defined as ν_icvf_ ≥ 0.999. **(A)** Sagittal, coronal, and axial view of the whole-brain SNR map (i), with a zoom-in view of the brainstem (ii). The brainstem is characterized by low SNR due to the spatial characteristics of the receive coil array (ii) and high occurrence of unrealistically high ν_icvf_ (iii), also shown as a binary mask (iv). **(B)** Given the co-occurrence of low SNR and unrealistically high ν_icvf_, a binary SNR mask was created to exclude low-SNR voxels. To determine the optimal threshold for the SNR mask, the ratio between the number of voxels with unrealistically high ν_icvf_ and the total number of voxels within the mask were plotted against the SNR threshold. The solid dots and error bars represent the group mean and group standard deviation of the ratio, respectively. The SNR value that yielded the minimum of this ratio was considered optimal (SNR = 39, shown in red).

A so-called high-SNR WM_*group*_ was also defined by taking the intersection of the WM_*group*_ and a binary signal-to-noise ratio (SNR) map. Hereby, the latter was used to reduce the number of voxels with unrealistically high values of ν_*icvf*_ (ν_icvf_≥0.999). In 6 of 25 subjects, an SNR map was created by dividing the mean b_0_ image by a single noise estimate in the native space and multiplied by the square root of the number of b_0_ images per DWI dataset (*n* = 12). The noise was estimated within a noise ROI outside the brain in 72 images (6 subjects, both timepoints and 6 b0 images each) using the ACID toolbox, with the values averaged to obtain a single noise estimate. The threshold for SNR maps to create binary SNR map was chosen such that it minimizes the ratio between the number of artifactual voxels where ν_icvf_≥0.999 and the total number of voxels in the SNR mask (Figure 3B), yielding a value of 39. This was motivated by the observation that unrealistically high ν_*icvf*_ values typically occur in low-SNR areas (Figures 3Aii,iii). This threshold selection represents a trade-off between removing unrealistic voxels while retaining as many voxels as possible.

### Region of Interest Selection

**FIGURE 4:**
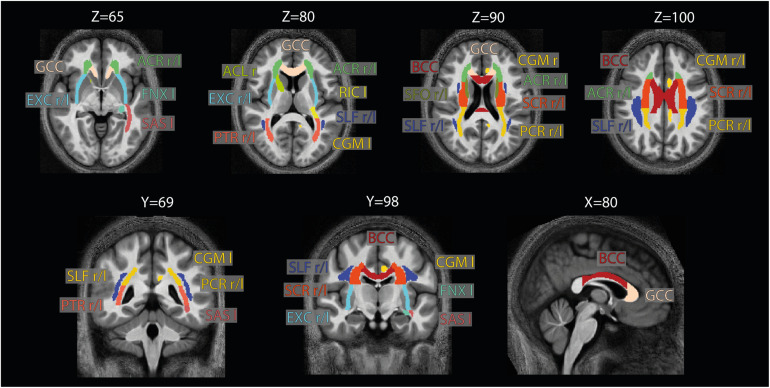
Location of the ROIs used for analysis. The 21 high-SNR ROIs (listed in Table 1) are part of the JHU-ICBM-DTI-81 WM atlas (Hua et al., 2008) and are displayed here on the group-averaged normalized ${\text{MT}}_{\text{sat}}^{\mathrm{B1}}$ image. Note that for ROI analysis, the ROIs were projected into the native space using the inverse of the combined deformation field.

For the region of interest (ROI) analysis, the JHU-ICBM-DTI-81 WM atlas (Hua et al., 2008) was transformed into the native space using the inverse of the combined deformation field. Two sets of ROIs were defined: (i) whole-WM ROIs and (ii) high-SNR ROIs, used for the main analysis. The whole-WM ROIs included those of the JHU-ICBM-DTI-81 WM atlas that were completely in WM_*group*_ defined in 2.6, yielding 43 ROIs (out of 48, leaving out the column and body of the fornix, the left and right cingulum part in the vicinity to the hippocampus, and the left and right uncinate fasciculus). The high-SNR ROIs included only those whole-WM ROIs that overlapped with the high-SNR WM_*group*_ to at least 95%, yielding 21 ROIs (Figure 4 and Table 2). For the analyses, group-averaged g_MR_, AVF_MR_, and MVF_MR_ were calculated within the WM_*group*_. Note that averaging included both sessions of each subject for all analyses except for the analysis in section “Test-Retest Analysis of the Group-Averaged MR G-ratio, Axon, and Myelin Volume Fraction.”

**TABLE 2:** Summary statistics of ${\text{g}}_{\text{MR}}^{\mathrm{B1}}$, ${\text{AVF}}_{\text{MR}}^{\mathrm{B1}}$, and ${\text{MVF}}_{\text{MR}}^{\mathrm{B1}}$.

	△_DR_	min_i∈ROI_	max_i∈ROI_	mean	SD
${\text{g}}_{\text{MR}}^{\mathrm{B1}}$,	0.046	0.642	0.688	0.664	0.014
${\text{AVF}}_{\text{MR}}^{\mathrm{B1}}$	0.076	0.308	0.384	0.337	0.020
${\text{MVF}}_{\text{MR}}^{\mathrm{B1}}$	0.037	0.408	0.445	0.425	0.010

*This table lists the dynamic range (△_DR_), lowest (min_i ∈ ROI_) and highest (max_i ∈ ROI_) ROI average value, mean value of the 21 analyzed ROI’s (mean) with its corresponding standard deviation (SD).*

### Test-Retest Analysis of the Group-Averaged MR G-ratio, Axon, and Myelin Volume Fraction

The group-averaged ${\text{g}}_{\text{MR}}^{\mathrm{B1}}$ of the first and second session were compared within the previously mentioned 21 high-SNR ROIs using Bland-Altman plots (Bland and Altman, 1986). In the Bland-Altmann plots, the differences in ${\text{g}}_{\text{MR}}^{\mathrm{B1}}$ between the first (${\text{g}}_{{\text{MR}}_{1}}^{\mathrm{B1}}$) and second (${\text{g}}_{{\text{MR}}_{2}}^{\mathrm{B1}}$) session ($\delta_{i}^{\text{retest}}={\left({\text{g}}_{{\text{MR}}_{1}}^{\mathrm{B1}}\right)}_{i}-{\left({\text{g}}_{{\text{MR}}_{2}}^{\mathrm{B1}}\right)}_{i}$) were plotted against their means (${\text{mean}}_{i}^{\text{retest}}=\frac{{\left({\text{g}}_{{\text{MR}}_{1}}^{\mathrm{B1}}\right)}_{i}+{\left({\text{g}}_{{\text{MR}}_{2}}^{\mathrm{B1}}\right)}_{i}}{2}$), where *i* is the index of ROI *i*. Bias captures the offset (${\bar{\delta }}^{\text{retest}}=\frac{1}{21}\sum_{i=1}^{21}\delta_{i}^{\text{retest}}$), while error ($\epsilon^{\text{retest}}=1.96\cdot \sqrt{\frac{1}{20}\sum_{i=1}^{21}\left(\delta_{i}^{\text{retest}}-{\bar{\delta }}^{\text{retest}}\right)}$) captures the variation between the first and second scan within the *i*^th^ ROI. The computed ${\bar{\delta }}^{\text{retest}}$ and ϵ^retest^ were normalized by the dynamic range (△_DR_) of ${\text{g}}_{\text{MR}}^{\mathrm{B1}}$ within the high-SNR ROIs, defined as ${△}_{\text{DR}}=\max_{\text{i}\in \text{ROI}}\left({\text{mean}}_{\text{i}}^{\text{retest}}\right)-\min_{\text{i}\in \text{ROI}}\left({\text{mean}}_{\text{i}}^{\text{retest}}\right)$, yielding the relative error ($\delta_{\text{DR}\%}^{\text{retest}}=\frac{\epsilon^{\text{retest}}}{{△}_{\text{DR}}}\cdot 100$) and relative bias (${\bar{\delta }}_{\text{DR}\%}^{\text{retest}}=\frac{{\bar{\delta }}^{\text{retest}}}{{△}_{\text{DR}}}\cdot 100$). *The same procedure was also applied to* ${\text{AVF}}_{\text{MR}}^{\mathrm{B1}}$ and ${\text{MVF}}_{\text{MR}}^{\mathrm{B1}}$.

The distinction between bias and error is important, because while a potential bias can be retrospectively corrected, the error in the MR g-ratio method defines its sensitivity to detect differences between individuals, groups, or time points. To reliably capture these differences, the error must be significantly lower than the expected effect size.

### Influence of B_1_+ Correction in the Group-Averaged MR G-ratio, Axon, and Myelin Volume Fraction

Bland-Altman analysis was used to compare g_MR_ with and without B_1_+ correction. In particular, the difference $\delta_{i}^{\text{B}1}$ in g_MR_ between ${\left(g_{\text{MR}}^{\mathrm{B1}}\right)}_{i}$, when using the reference method B_1_+ correction, and ${\left(g_{\text{MR}}^{\text{k}}\right)}_{i}$, when using no (k = NO) or UNICORT (k = UN) B_1_+ correction: $\delta_{i}^{\text{B}1}={\left(g_{\text{MR}}^{\mathrm{B1}}\right)}_{i}-{\left(g_{\text{MR}}^{\text{k}}\right)}_{i}$ was plotted against their mean: ${\text{mean}}_{i}^{\mathrm{B1}}=\frac{{\left(g_{\text{MR}}^{\mathrm{B1}}\right)}_{i}+{\left({\text{g}}_{\text{MR}}^{\text{k}}\right)}_{i}}{2}$, with *i* being the index of the 21 high-SNR ROIs. The bias and error associated with the lack of (or UNICORT) B_1_+ correction are defined as ${\bar{\delta }}^{\mathrm{B1}}=\frac{1}{21}\sum_{i=1}^{21}\delta_{i}^{\mathrm{B1}}$ and $\epsilon^{\mathrm{B1}}=1.96\cdot \sqrt{\frac{1}{20}\sum_{i=1}^{21}\left(\delta_{i}^{\mathrm{B1}}-{\bar{\delta }}^{\mathrm{B1}}\right)}$, respectively.

The computed ϵ^B1^ and ${\bar{\delta }}^{\mathrm{B1}}$were normalized by the dynamic range of ${\text{g}}_{\text{MR}}^{\mathrm{B1}}$ within the high-SNR ROIs, yielding the relative error ($\epsilon_{\text{DR}\%}^{\mathrm{B1}}=\frac{\epsilon^{\mathrm{B1}}}{{△}_{\text{DR}}}\cdot 100$) and relative bias (${\bar{\delta }}_{\text{DR}\%}^{\mathrm{B1}}=\frac{{\bar{\delta }}^{\mathrm{B1}}}{{△}_{\text{DR}}}\cdot 100$). The same procedure was also applied to AVF_MR_ and MVF_MR_, comparing them to their respective reference method and dynamic range. For MVF_MR_, the Bland-Altman analysis was additionally done using the whole-WM ROIs instead of the high-SNR ROIs (see section “Region of Interest Selection”) to assess the influence of including low-SNR voxels in the analysis.

### Group Variability in MR G-ratio, Axon, and Myelin Volume Fraction

To assess group variability for each correction method, the coefficient-of-variation (CoV) across subjects and sessions was calculated for MVF_MR_, AVF_MR_, and g_MR_ in the MNI space after applying tissue-weighted smoothing (Tabelow et al., 2019), yielding: ${\text{CoV}}_{\text{MR}}^{\mathrm{B1}}$, ${\text{CoV}}_{\text{MR}}^{\text{UN}}$, and ${\text{CoV}}_{\text{MR}}^{\text{NO}}$, where MR ∈ {g_MR_,AVF_MR_,andMVF_MR_}. For tissue-weighted smoothing, a full width at half maximum Gaussian smoothing kernel of 6 mm was used. Bland-Altman analysis (see section “Test-Retest Analysis of the Group-Averaged MR G-ratio, Axon, and Myelin Volume Fraction”) was used to compare ${\text{CoV}}_{\text{MR}}^{\text{UN}}$ and ${\text{CoV}}_{\text{MR}}^{\text{NO}}$ against ${\text{CoV}}_{\text{MR}}^{\mathrm{B1}}$ based on the reference method, yielding bias (${\bar{\delta }}^{\text{CoV}}$) and error (ϵ^CoV^) values. A higher variability across the brain is expected to increase ${\bar{\delta }}^{\text{CoV}}$ whereas a higher local variability is expected to increase ϵ^ CoV^.

## Results

### G-ratio, Myelin, and Axonal Volume Fraction Across the White Matter

**FIGURE 5:**
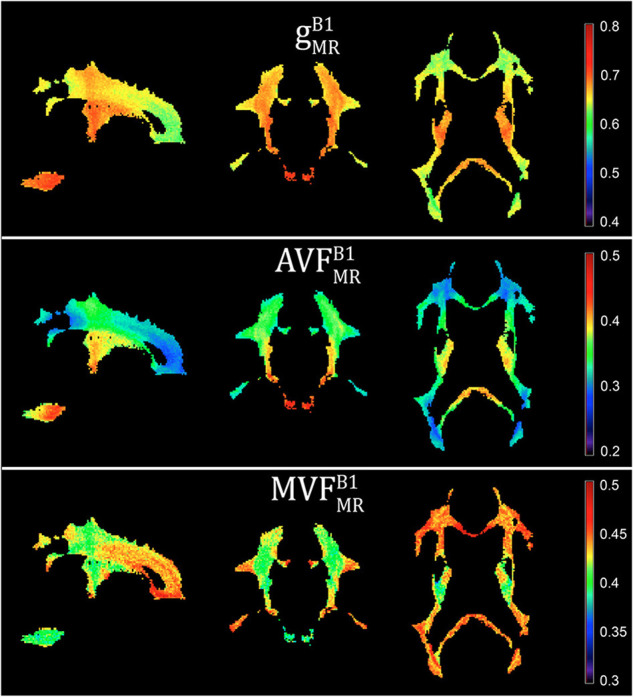
Voxel-wise maps of group-averaged g^*B1*^_MR_, AVF^*B1*^_MR_, and MVF^*B1*^_MR_, restricted to the group WM mask (cf. section “Definition of White Matter Masks”). Depicted are a single sagittal (x = 100), coronal (y = 91), and axial (z = 85) slice.

**FIGURE 6:**
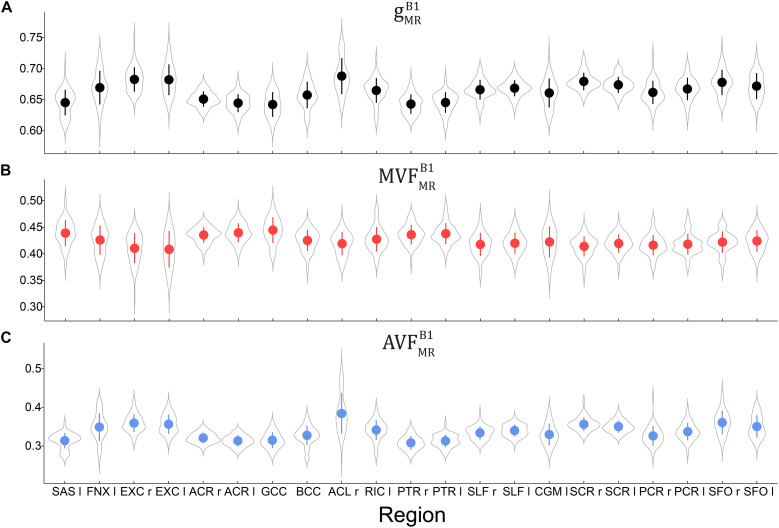
Violin plots representing the distribution of ${\text{g}}_{\text{MR}}^{\mathrm{B1}}$ **(A)**, ${\text{MVF}}_{\text{MR}}^{\mathrm{B1}}$ **(B)**, and ${\text{AVF}}_{\text{MR}}^{\mathrm{B1}}$ **(C)** across the group and in 21 high-SNR ROIs listed in Table 1. The mean and standard deviation of the distribution are indicated by solid dot and whiskers, respectively.

Voxel-wise maps of group-averaged ${\text{g}}_{\text{MR}}^{\mathrm{B1}}$, ${\text{AVF}}_{\text{MR}}^{\mathrm{B1}}$, and ${\text{MVF}}_{\text{MR}}^{\mathrm{B1}}$ in WM are shown in Figure 5. The group-averaged mean and standard deviation of ${\text{g}}_{\text{MR}}^{\mathrm{B1}}$, ${\text{MVF}}_{\text{MR}}^{\mathrm{B1}}$, and ${\text{AVF}}_{\text{MR}}^{\mathrm{B1}}$ in 21 high-SNR ROIs are reported in Table 1 and Figure 6. The dynamic range (△_DR_), minimum and maximum values, and mean and standard deviation of ${\text{g}}_{\text{MR}}^{\mathrm{B1}}$, ${\text{AVF}}_{\text{MR}}^{\mathrm{B1}}$, and ${\text{MVF}}_{\text{MR}}^{\mathrm{B1}}$ across ROIs are listed in Table 2. The largest ${\text{g}}_{\text{MR}}^{\mathrm{B1}}$ and ${\text{AVF}}_{\text{MR}}^{\mathrm{B1}}$ were found in the right anterior limb of the internal capsule (0.688 and 0.384, respectively), while the largest ${\text{MVF}}_{\text{MR}}^{\mathrm{B1}}$ was in the genu of corpus callosum (0.445), where also the lowest ${\text{g}}_{\text{MR}}^{\mathrm{B1}}$ (0.642) can be found. The lowest ${\text{AVF}}_{\text{MR}}^{\mathrm{B1}}$, and ${\text{MVF}}_{\text{MR}}^{\mathrm{B1}}$ were found in the right posterior thalamic radiation (${\text{AVF}}_{\text{MR}}^{\mathrm{B1}}$ = 0.308) and in the left external capsule (${\text{MVF}}_{\text{MR}}^{\mathrm{B1}}$ = 0.408), respectively. The △_*DR*_ was the smallest for ${\text{MVF}}_{\text{MR}}^{\mathrm{B1}}$ (0.037), followed by ${\text{g}}_{\text{MR}}^{\mathrm{B1}}$ (0.046) and ${\text{AVF}}_{\text{MR}}^{\mathrm{B1}}$ (0.076).

### Test-Retest Analysis of the Group-Averaged MR G-ratio, Axon, and Myelin Volume Fraction

**FIGURE 7:**
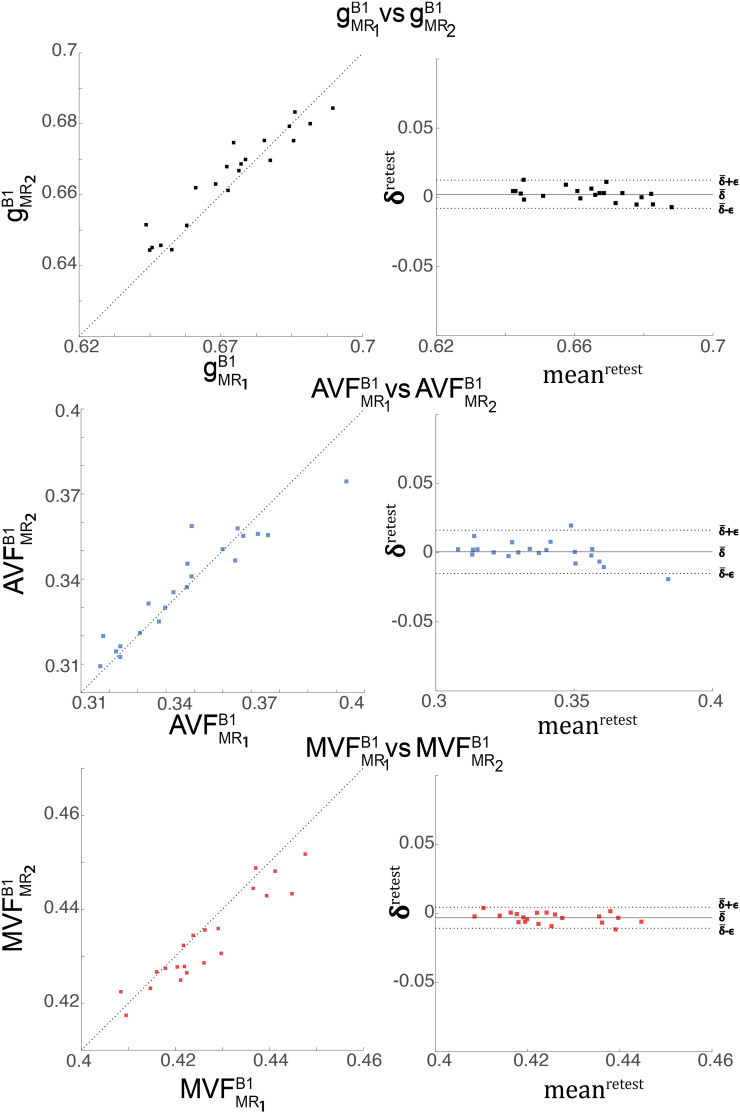
Depicted are scatter and Bland-Altman plots of ${\text{g}}_{\text{MR}}^{\mathrm{B1}}$ (first row), ${\text{AVF}}_{\text{MR}}^{\mathrm{B1}}$ (second row), and ${\text{MVF}}_{\text{MR}}^{\mathrm{B1}}$(third row) from two session across 21 WM regions (denoted high-SNR ROIs, see Figure 4). The Bland-Altman plot illustrates the differences between values obtained from the two sessions (e.g., ${\text{g}}_{{\mathrm{MR}}_{1}}^{\mathrm{B1}}$ vs. ${\text{g}}_{\mathrm{MR2}}^{\mathrm{B1}}$; $\delta_{i}^{\text{retest}}={\left({\text{g}}_{\mathrm{MR1}}^{\mathrm{B1}}\right)}_{i}-{\left({\text{g}}_{\mathrm{MR2}}^{\mathrm{B1}}\right)}_{i}$) against their mean (e.g., ${\text{mean}}_{i}^{\text{retest}}=\frac{{\left({\text{g}}_{\mathrm{MR1}}^{\mathrm{B1}}\right)}_{i}+{\left({\text{g}}_{\mathrm{MR2}}^{\mathrm{B1}}\right)}_{i}}{2}$, with *i* indexing the *i*^th^ ROI). Each point in the scatter plot represents the group-averaged value in a single ROI. The bold black line represents the bias (${\bar{\delta }}^{\text{retest}}=\frac{1}{21}\sum_{i=1}^{21}\delta_{i}^{\text{retest}}$), while the dashed line shows error ($\epsilon^{\text{retest}}=1.96\cdot \mathrm{SD}\left(\delta_{i}^{\text{retest}}\right)$) between the two sessions.

The relative error ($\epsilon_{\text{DR}\%}^{\text{retest}}$) and bias (${\bar{\delta }}_{\text{DR}\%}^{\text{retest}}$) values of the test-retest analysis are summarized in Table 3 and shown as Bland-Altmann plots in Figure 7. The test-retest analysis revealed a ${\bar{\delta }}_{\text{DR}\%}^{\text{retest}}$ below an absolute value of 8.4% for each metric (${\text{g}}_{\text{MR}}^{\mathrm{B1}}$, ${\text{AVF}}_{\text{MR}}^{\mathrm{B1}}$, and ${\text{MVF}}_{\text{MR}}^{\mathrm{B1}}$), where the ${\text{AVF}}_{\text{MR}}^{\mathrm{B1}}$ showed the lowest ${\bar{\delta }}_{\text{DR}\%}^{\text{retest}}$ with 0.79% (Figure 7 and Table 3). The $\epsilon_{\text{DR}\%}^{\text{retest}}$ was below 22.2% for each metric, where the ${\text{AVF}}_{\text{MR}}^{\mathrm{B1}}$ showed the lowest $\epsilon_{\text{DR}\%}^{\text{retest}}$ with 20.5% (Figure 7 and Table 3).

**TABLE 3:** Bias and error between scans, in ${\text{g}}_{\text{MR}}^{\mathrm{B1}}$, ${\text{AVF}}_{\text{MR}}^{\mathrm{B1}}$, and ${\text{MVF}}_{\text{MR}}^{\mathrm{B1}}$.

MAP	${\bar{\delta }}^{\text{retest}}$	ϵ^retest^	${\bar{\delta }}_{\text{DR}\%}^{\text{retest}}$	$\epsilon_{\text{DR}\%}^{\text{retest}}$
${\text{g}}_{\text{MR}}^{\mathrm{B1}}$	0.0021	0.0102	4.57	22.17
${\text{AVF}}_{\text{MR}}^{\mathrm{B1}}$	0.0006	0.0156	0.79	20.53
${\text{MVF}}_{\text{MR}}^{\mathrm{B1}}$	−0.0031	0.0076	−8.38	20.54

*List of the bias (${\bar{\delta }}^{retest}$) and error (ϵ^retest^) values, defined as in Figure 7, along with their relative value with respect to the dynamic range △_DR_: $\epsilon_{DR\%}^{retest}=\frac{\epsilon^{retest}}{{△}_{DR}}\cdot 100$; ${\bar{\delta }}_{DR\%}^{retest}=\frac{{\bar{\delta }}^{retest}}{{△}_{DR}}\cdot 100$.*

### Influence of B_1_+ Correction on the Group-Averaged MR G-ratio, Axon, and Myelin Volume Fraction

**FIGURE 8:**
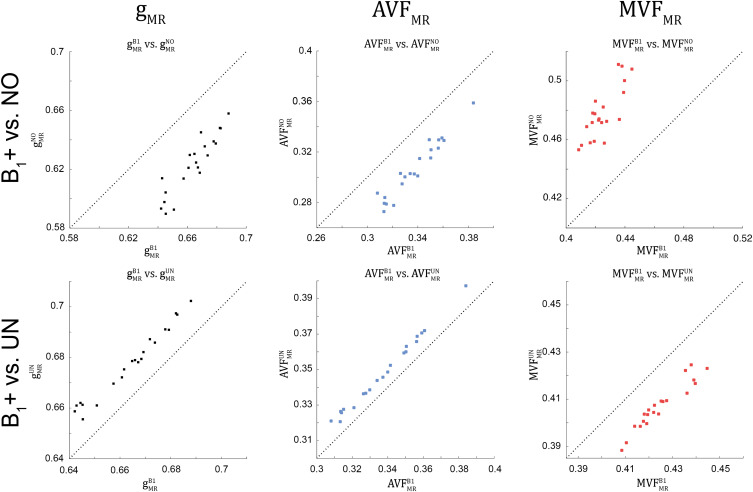
Scatter plots of g_MR_, AVF_MR_, and MVF_MR_, plotting values obtained without B_1_+ correction (superscript: NO, top row) and with UNICORT B_1_+ correction (superscript: UN, bottom row) against values obtained with the reference method, i.e., B_1_+ field map correction (superscript: B1). A dashed unit slope line is plotted for reference. Each point in the scatter plot represents the group-averaged value in a single ROI (see Figure 4 for the locations of the 21 high-SNR ROIs).

**FIGURE 9:**
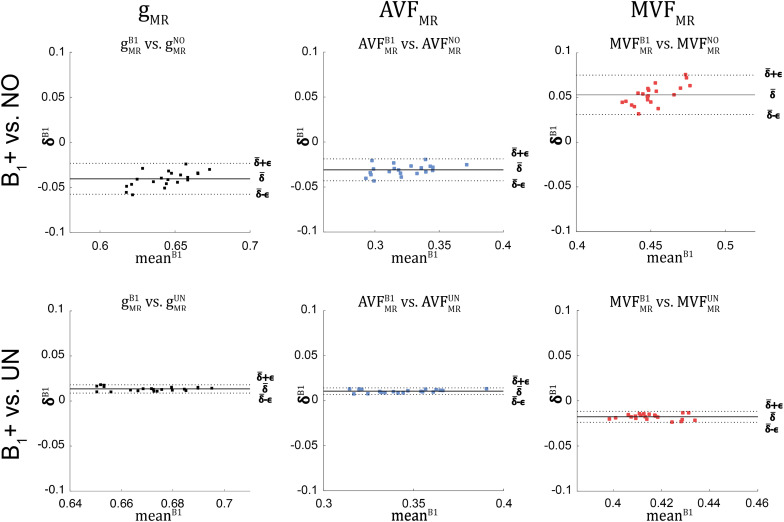
Bland-Altman plots of g_MR_, AVF_MR_, and MVF_MR_, comparing values obtained without B_1_+ correction (NO, top row) and with UNICORT B_1_+ correction (UN, bottom row) against values obtained by B_1_+ field map correction (superscript: B1). The Bland-Altman plot illustrates the differences between values obtained by two different methods (reference vs. tested method); e.g., $\delta_{i}^{\mathrm{B1}}={\left(g_{\text{MR}}^{\mathrm{B1}}\right)}_{i}-{\left(g_{\text{MR}}^{\text{k}}\right)}_{i}$ against their mean (${\text{mean}}_{i}^{\mathrm{B1}}=\frac{{\left(g_{\text{MR}}^{\mathrm{B1}}\right)}_{i}+{\left({\text{g}}_{\text{MR}}^{\text{k}}\right)}_{i}}{2}$, with k = {*UN*, *NO*} and *i* indexing the *i*^th^ ROI). Each point in the scatter plot represents the group-averaged value in a single ROI (see Figure 4 for the locations of the 21 high-SNR ROIs). The bold black line represents the bias (${\bar{\delta }}^{\mathrm{B1}}=\sum_{i=1}^{21}\delta_{i}^{\mathrm{B1}}$), while the dashed line shows error ($\epsilon^{\mathrm{B1}}=1.96\cdot \mathrm{SD}\left(\delta_{i}^{\mathrm{B1}}\right)$) between the reference and the tested method. Error and bias values averaged across all ROIs and subjects are listed in Table 5.

The relative error ($\epsilon_{\text{DR}\%}^{\mathrm{B1}}$) and bias (${\bar{\delta }}_{\text{DR}\%}^{\mathrm{B1}}$) values of the B_1_+ correction analysis are summarized in Table 4 and shown as Bland-Altmann plots in Figures 8, 9. For g_MR_, compared to the no-correction case, UNICORT showed both lower $\epsilon_{\text{DR}\%}^{\mathrm{B1}}$ (UNICORT vs. no correction: 10.9% vs. 37.0%) and ${\bar{\delta }}_{\text{DR}\%}^{\mathrm{B1}}$ (30.4% vs. −89.1%). For both AVF_MR_ and MVF_MR_, UNICORT yielded lower $\epsilon_{\text{DR}\%}^{\mathrm{B1}}$ (UNICORT vs. no correction; AVF_MR_: 5.3% vs. 15.8%; 16.2% vs. 59.5%) and lower ${\bar{\delta }}_{\text{DR}\%}^{\mathrm{B1}}$ (AVF_MR_: 14.5% vs. −40.8%; MVF_MR_: 48.6% vs. 143.2%). Altogether, the UNICORT correction reduced the bias and error in the MR g-ratio and its constituents by roughly a factor of three. The lower $\epsilon_{\text{DR}\%}^{\mathrm{B1}}$ and ${\bar{\delta }}_{\text{DR}\%}^{\mathrm{B1}}$ associated with UNICORT was also reflected by the fact that values of ${\text{g}}_{\text{MR}}^{\text{UN}}$, ${\text{AVF}}_{\text{MR}}^{\text{UN}}$, and ${\text{MVF}}_{\text{MR}}^{\text{UN}}$ (Figure 8, lower panel) lie closer to the unit slope line than values of ${\text{g}}_{\text{MR}}^{\text{NO}}$, ${\text{AVF}}_{\text{MR}}^{\text{NO}}$, and ${\text{MVF}}_{\text{MR}}^{\text{NO}}$ (Figure 8, upper panel). When computing $\epsilon_{\text{DR}\%}^{\mathrm{B1}}$ and ${\bar{\delta }}_{\text{DR}\%}^{\mathrm{B1}}$ of g_MR_ in the whole-WM ROIs (see Supplementary Figure 1), ${\bar{\delta }}_{\text{DR}\%}^{\mathrm{B1}}$ was consistently lower for both the no-correction case (whole-WM ROIs vs. high-SNR ROIs: 36.5% vs. 143.2%) and UNICORT (13.1% vs. 48.6%), whereas $\epsilon_{\text{DR}\%}^{\mathrm{B1}}$ was similar (no-correction: 52.8% vs. 59.5%; UNICORT: 24.0% vs. 16.2%).

**TABLE 4:** Bias and error between methods, in g_MR_, AVF_MR_, and MVF_MR_.

MAP	${\bar{\delta }}^{\mathrm{B1}}$	ϵ^B1^	${\bar{\delta }}_{\text{DR}\%}^{\mathrm{B1}}$	$\epsilon_{\text{DR}\%}^{\mathrm{B1}}$
${\text{g}}_{\text{MR}}^{\mathrm{B1}}$ vs. ${\text{g}}_{\text{MR}}^{\text{NO}}$	–0.041	0.017	–89.13	36.96
${\text{g}}_{\text{MR}}^{\mathrm{B1}}$ vs. ${\text{g}}_{\text{MR}}^{\text{UN}}$	0.014	0.005	30.44	10.87
${\text{AVF}}_{\text{MR}}^{\mathrm{B1}}$ vs. ${\text{AVF}}_{\text{MR}}^{\text{NO}}$	–0.031	0.012	–40.79	15.79
${\text{AVF}}_{\text{MR}}^{\mathrm{B1}}$ vs. ${\text{AVF}}_{\text{MR}}^{\text{UN}}$	0.011	0.004	14.47	5.26
${\text{MVF}}_{\text{MR}}^{\mathrm{B1}}$ vs. ${\text{MVF}}_{\text{MR}}^{\text{NO}}$	0.053	0.022	143.24	59.46
${\text{MVF}}_{\text{MR}}^{\mathrm{B1}}$ vs. ${\text{MVF}}_{\text{MR}}^{\text{UN}}$	–0.018	0.006	–48.65	16.22
EWM ${\text{MVF}}_{\text{MR}}^{\mathrm{B1}}$ vs. ${\text{MVF}}_{\text{MR}}^{\text{NO}}$	0.033	0.048	36.48	52.75
EWM ${\text{MVF}}_{\text{MR}}^{\mathrm{B1}}$ vs. ${\text{MVF}}_{\text{MR}}^{\text{UN}}$	–0.012	0.022	–13.08	23.96

*List of the bias (${\bar{\delta }}^{B1}$) and error (ϵ^B1^) values as defined in Figure 9, along with their relative value with respect to the dynamic range△_DR_: $\epsilon_{DR\%}^{B1}=\frac{\epsilon^{B1}}{{△}_{DR}}\cdot 100$; ${\bar{\delta }}_{DR\%}^{B1}=\frac{{\bar{\delta }}^{B1}}{{△}_{DR}}\cdot 100$. Note that the error and bias in the last two rows were obtained when using the whole-WM ROIs instead of the high-SNR ROIs (see Supplementary Figure 1).*

### Group Variability in MR G-ratio, Axon, and Myelin Volume Fraction

**FIGURE 10:**
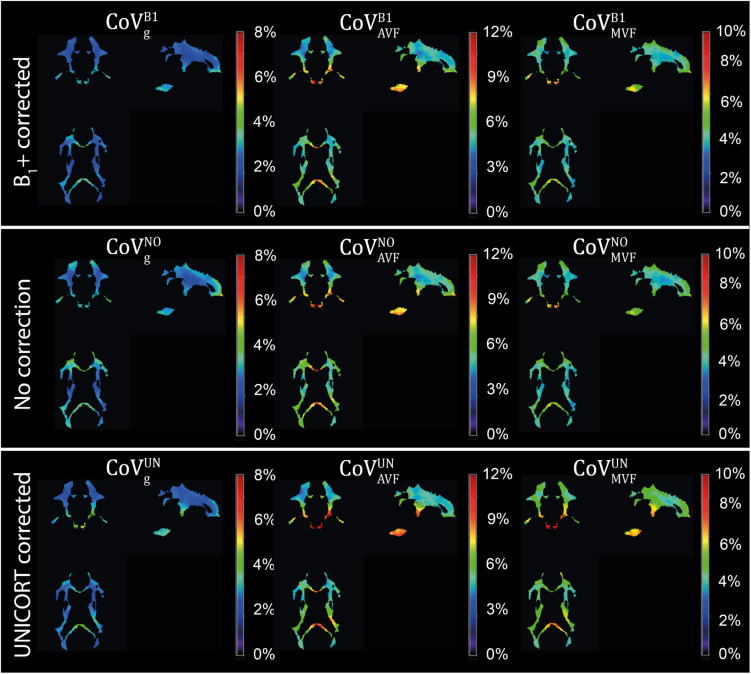
Coefficient of variation (CoV) maps of g_MR_, AVF_MR_, and MVF_MR_ with B_1_+ correction (${\text{CoV}}_{\text{g}}^{\mathrm{B1}}$, ${\text{CoV}}_{\text{AVF}}^{\mathrm{B1}}$, and ${\text{CoV}}_{\text{MVF}}^{\mathrm{B1}}$), no correction (${\text{CoV}}_{\text{g}}^{\text{NO}}$, ${\text{CoV}}_{\text{AVF}}^{\text{NO}}$, and ${\text{CoV}}_{\text{MVF}}^{\text{NO}}$), and UNICORT B_1_+ correction (${\text{CoV}}_{\text{g}}^{\text{UN}}$, ${\text{CoV}}_{\text{AVF}}^{\text{UN}}$, and ${\text{CoV}}_{\text{MVF}}^{\text{UN}}$). CoV maps, expressed in percentage, were computed as the voxel-wise ratio between the group mean and group standard deviation maps of the normalized g_MR_, AVF_MR_, or MVF_MR_. The voxel-wise computation of CoV is restricted to the group WM mask (cf. section “Definition of White Matter Masks”). Shown are a single coronal (y = 91), sagittal (x = 100), and axial (z = 85) slice.

**FIGURE 11:**
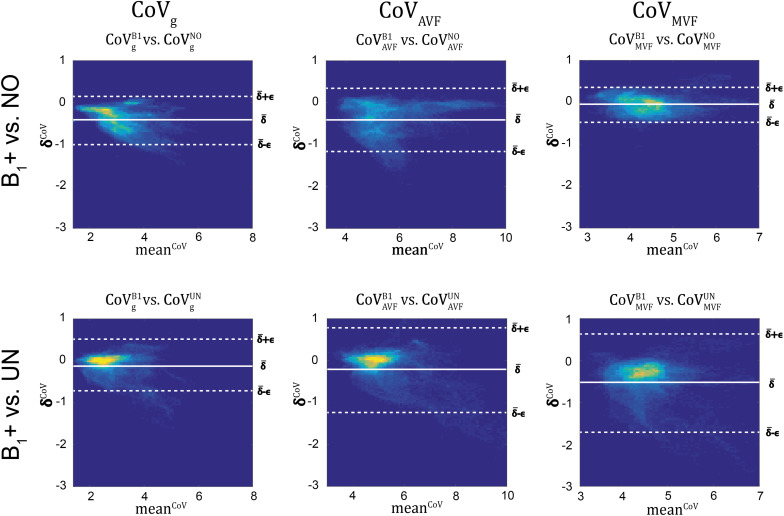
Bland-Altman density plots of CoV_g_, CoV_AVF_, and CoV_MVF_ for no correction (NO, top row) and UNICORT B_1_+ correction (UN, bottom row) against the reference method (B_1_+) (yellow indicates high density and blue low). The Bland-Altman plot depicts the differences between the tested parameter maps and the reference method (e.g., $\delta_{i}^{\text{CoV}}={\left({\mathrm{CoV}}_{\text{g}}^{\mathrm{B1}}\right)}_{i}-{\left({\mathrm{CoV}}_{\text{g}}^{\text{k}}\right)}_{i}$) against their mean (e.g., ${\text{mean}}_{i}^{\text{CoV}}=\frac{{\left({\mathrm{CoV}}_{\text{g}}^{\mathrm{B1}}\right)}_{i}+{\text{CoV}}_{\text{g}}^{\text{k}}){}_{i}}{2}$) with k = {*UN*,*NO*} and *i* being the index of the *i*^th^ region. The bold white line represents the bias (${\bar{\delta }}^{\text{CoV}}=\sum_{i=1}^{\text{N}}{}_{i}^{\text{CoV}}$; N = number of voxels) and the dashed lines represent ${\bar{\delta }}^{\text{CoV}}$ the error ($\epsilon^{\text{CoV}}=1.96\cdot \mathrm{SD}\left(\delta_{\text{i}}^{\text{CoV}}\right)$]. The error and bias values are summarized in Table 5.

**FIGURE 12:**
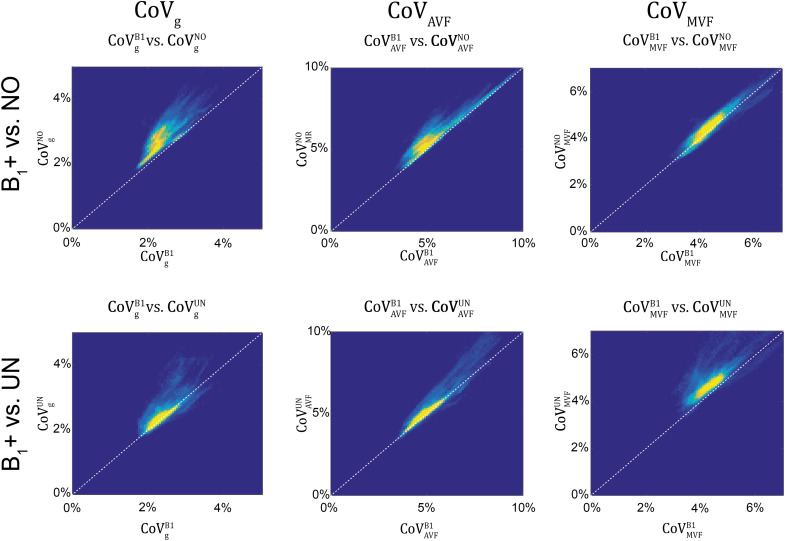
Scatter density plots of CoV_g_ (left column), CoV_AVF_ (middle column), and CoV_MVF_ (right column), plotting values obtained with no correction (superscript: NO, top row) and with UNICORT B_1_+ correction (UN, bottom row) against values obtained by B_1_+ field map correction (superscript: B1). The unit slope line is plotted for orientation (dotted line). The dots in the scatter plots represent the WM voxels in the CoV maps in Figure 10 (yellow indicates high voxel density).

g_MR_ showed on average smaller CoV than AVF_MR_ and MVF_MR_ (Figure 10). In all maps, the CoV was the highest in the deep brain areas. The relative error ($\frac{\epsilon^{\text{CoV}}}{{\text{CoV}}^{\mathrm{B1}}}\cdot 100$) and bias ($\frac{{\bar{\delta }}^{\text{CoV}}}{{\text{CoV}}^{\mathrm{B1}}}\cdot 100$) values of CoV with respect to the B_1_+ reference measurement are summarized in Table 5 and the error and bias are also displayed as Bland-Altman density plot in Figure 11. For g_MR_, compared to the no correction case, UNICORT showed similar ϵ^CoV^ (UNICORT vs. no correction: 0.6% vs. 0.6%) but lower ${\bar{\delta }}^{\text{CoV}}$ (−0.1% vs. −0.4%). UNICORT yielded higher ϵ^CoV^ (UNICORT vs. no correction; 1.0% vs. 0.8%) and lower ${\bar{\delta }}^{\text{CoV}}$ (−0.2% vs. −0.4%) for AVF_MR_, and higher ϵ^CoV^ (1.2% vs. 0.4%) and higher ${\bar{\delta }}^{\text{CoV}}$(−0.5% vs. −0.1%) for MVF_MR_. The lower ${\bar{\delta }}^{\text{CoV}}$ of g_MR_ and AVF_MR_ associated with UNICORT reveals itself as a slight shift of the points toward the unit slope line in the scatter density plot (Figure 12).

**TABLE 5:** Bias and error between methods, in the CoV of g_MR_, AVF_MR_, and MVF_MR_.

MAP	${\bar{\delta }}^{\text{CoV}}$	ϵ^CoV^	$\frac{{\bar{\delta }}^{\text{CoV}}}{{\text{CoV}}_{\text{MR}}^{B1}}\cdot 100$	$\frac{\epsilon^{\text{CoV}}}{{\text{CoV}}_{\text{MR}}^{B1}}\cdot 100$
CoV ${\text{g}}_{\text{MR}}^{\mathrm{B1}}$ vs. CoV ${\text{g}}_{\text{MR}}^{\text{NO}}$	−0.42	0.56	−17.3	23.1
CoV ${\text{g}}_{\text{MR}}^{\mathrm{B1}}$ vs. CoV ${\text{g}}_{\text{MR}}^{\text{UN}}$	−0.12	0.62	−4.9	25.5
CoV ${\text{AVF}}_{\text{MR}}^{\mathrm{B1}}$ vs. CoV ${\text{AVF}}_{\text{MR}}^{\text{NO}}$	−0.40	0.78	−7.3	14.3
CoV ${\text{AVF}}_{\text{MR}}^{\mathrm{B1}}$ vs. CoV ${\text{AVF}}_{\text{MR}}^{\text{UN}}$	−0.21	1.02	−3.8	18.7
CoV ${\text{MVF}}_{\text{MR}}^{\mathrm{B1}}$ vs. CoV ${\text{MVF}}_{\text{MR}}^{\text{NO}}$	−0.05	0.41	−1.1	9.2
CoV ${\text{MVF}}_{\text{MR}}^{\mathrm{B1}}$ vs. CoV ${\text{MVF}}_{\text{MR}}^{\text{UN}}$	−0.52	1.20	−11.9	27.0

*List of the bias (${\bar{\delta }}^{CoV}$) and error (ϵ^CoV^) values as defined in Figure 11, along with their relative value with respect to the group-average CoV across the MR g-ratios using the reference B_1_+ field correction method:$\frac{{\bar{\delta }}^{CoV}}{CoV_{MR}^{B1}}\cdot 100$;$\frac{\epsilon^{CoV}}{CoV_{MR}^{B1}}\cdot 100$.*

## Discussion

In this study, we showed that omitting the correction of the magnetization transfer saturation map (MT_sat_) for residual B_1_ + effects introduces large error and bias in the MR g-ratio and the constituents (myelin and axon volume fractions, or in short MVF_MR_ and AVF_MR_). We also demonstrated that this error and bias can be reduced by roughly a factor of three using the data-driven UNICORT B_1_+ correction (implemented in the hMRI toolbox, see text footnote 1) when a B_1_+ field measurement is unavailable.

### The Effect of Omitting the B_1_+ Field Measurement

MT_sat_ have been often used as a proxy for the MVF_MR_ in g-ratio weighted imaging (Mohammadi et al., 2015; Campbell et al., 2018; Ellerbrock and Mohammadi, 2018; Hori et al., 2018; Kamagata et al., 2019), because they are directly linked to the macromolecular pool with an intrinsic correction for underlying longitudinal relaxation time and B_1_+ field inhomogeneities effects (Helms et al., 2008). Despite the latter intrinsic correction for B_1_+ field inhomogeneities, we found that the residual B_1_+ effects on MT_sat_ map were still observable. In particular, the bias and error of the MR g-ratio (g_MR_) was about −89 and 37% higher, respectively, when omitting the B_1_+ correction. We found the same trend for MVF_MR_ and AVF_MR_; while the error and bias were even larger for MVF_MR_ when B_1_+ correction was omitted, it was smaller but still substantial for the AVF_MR_. We found that omitting B_1_ + leads to a substantially higher (more than 10-fold) bias in the MR g-ratio and its constituents when compared to a test-retest analysis of our data (Figure 7 and Table 3). Also, the error due to omitting the B1+ correction was twice as large as the error observed in the test retest analysis for the MR g-ratio and the MVF, whereas for AVF the errors were similar. We expect that the high error will be of particular relevance for group studies because it can be regarded as an error that evolves when replacing the reference method with the alternative method. For comparison, age-related changes assessed by g-ratio weighted imaging (Cercignani et al., 2017; Berman et al., 2018) have been reported to vary between 30 and 100% (in absolute values: g_MR_0.02–0.04 (Figure 5 in Cercignani et al., 2017). Consequently, the reported effect size of age-related changes would have become potentially undetectable if the B_1_+ field correction has been omitted in the study of Cercignani et al. (2017). The B_1_+ effect is particularly relevant for the MR g-ratio method by Cercignani et al. (2017) that combined quantitative MT (Gloor et al., 2008) with NODDI, because the qMT method does not possess an intrinsic correction for B_1_+ field inhomogeneities as opposed to the MT_sat_ methods used here. Note that we reported, for better intuition, the bias and error relative to the dynamic range of the parameters across the investigated white matter (WM) ROIs (the dynamic range of g_MR_ is △_DR_ = 0.046; the absolute bias and error can be found in Table 4).

To reduce this source of bias and error, we propose a data-driven approach to correct for B_1_+ field inhomogeneities when no B_1_+ field measurement is available. To this end, we used UNICORT to estimate the B_1_+ field (Weiskopf et al., 2011). We found that using the UNICORT-estimated B_1_+ field to correct residual B_1_+ field inhomogeneities in MT_sat_ reduces at the group level the bias and error in the MR g-ratio and its constituents by roughly a factor of three. However, the UNICORT estimated B_1_+ inhomogeneity can be erroneous with the error varying across subjects. To assess this variability, we estimated coefficient-of-variance (CoV) maps of g_MR_, AVF_MR_, and MVF_MR_ for all three methods. In general, an increased CoV can be found at tissue boundaries (e.g., cerebral spinal fluid to WM) due to slight misregistration between the maps of axonal and myelin markers and/or imperfect normalization (Figure 10). Additionally, we found a strong increase in the bias and error of the CoV of MVF maps (increase in bias: 11% and in error: 18%) when UNICORT B_1_+ correction was used as compared to no correction. The CoV of g_MR_ and AVF_MR_ did not show a consistent trend: while the bias decreased, the error increased for both parameters. In other words, the UNICORT B_1_+ correction leads to higher accuracy in the g-ratio and its constituents but comes at the cost of a lower precision in MVF.

### G-ratio, Myelin, and Axonal Volume Fraction Across the White Matter

Our ${\text{g}}_{\text{MR}}^{\mathrm{B1}}$ and ${\text{AVF}}_{\text{MR}}^{\mathrm{B1}}$ across the white matter were within the range of the reported values of previous studies (g_MR_: 0.64–0.76; AVF_MR_: 0.26–0.43 in (Cercignani et al., 2017; Berman et al., 2018). The range of ${\text{MVF}}_{\text{MR}}^{\mathrm{B1}}$ was in the upper half of previously reported values (0.17–0.42 in Cercignani et al., 2017). Our slightly higher MVF_MR_ values might be due to differences in the calibration approach: while we calculated the reference MVF_REF_ from previously published *ex-vivo* histology data (Graf von Keyserlingk and Schramm, 1984), Cercignani et al. (2017), used a reference from previously published *ex-vivo* histology g-ratio data in the corpus callosum and Berman et al. (2018), did not perform any calibration assuming that macromolecular tissue volume and MVF_MR_ are equal. An error in the calibration constant can lead to a bias in the MVF estimates which in turn leads to an error and bias in the MR g-ratio (Campbell et al., 2018).

### Confounding Factors

As this study calculates the *in-vivo* MR g-ratio, there is no histological data available from the participants of this study, which could be used for calibration or as a gold standard reference. For calibration of MT_sat_ to MVF_MR_, we estimated the histological MVF (MVF_hist_) from published *ex-vivo* data within the human medulla oblongata (Graf von Keyserlingk and Schramm, 1984). Since the reference MVF_hist_ and the calibrated MT_sat_ map were taken from different subjects, this might introduce a systematic bias in the MR g-ratio. However, since we found a relatively good agreement between our g_MR_, AVF_MR_, and MVF_MR_ values with previously reported values obtained by a different calibration approach (Cercignani et al., 2017; Berman et al., 2018), we expect that it had a small effect on the results. Moreover, we focused on the effect of omitting B_1_+ correction, which will lead to additional inaccuracies in g-ratio weighted imaging, independent of the quality of the calibration.

Although, not reported in previous NODDI-based g-ratio mapping studies (Stikov et al., 2015; Cercignani et al., 2017; Jung et al., 2017; Mancini et al., 2017; Ellerbrock and Mohammadi, 2018; Hori et al., 2018), we found that the intra-cellular volume fraction (ν_*icvf*_) determined with NODDI tends to be biased at small signal-to-noise ratios (SNR < 39), resulting in a ceiling effect, i.e., ν_icvf_ ≈ 1. To avoid a corresponding bias in g_MR_ (and AVF_MR_), we restricted the analysis to regions with sufficiently high SNR (Figure 3). To investigate whether our findings generalize to low-SNR regions as well, we performed an additional Bland-Altman analysis of MVF_MR_ in whole-WM ROIs. To this end, a larger set of ROIs was used covering the entire white matter. Although the bias was smaller for the whole-WM as compared to the high-SNR ROI analysis, we found the same trend: the error and bias were reduced when using UNICORT B_1_+ correction relative to no correction. Note that the smaller bias for the whole-WM analysis is most probably an artifact of the calibration procedure. Since the ROI used for calibration was not part of the high-SNR ROIs but was part of the whole-WM ROIs, we think it could have reduced the bias in the whole-WM ROI analysis as compared to the high-SNR analysis.

We note that the presented results were based on a customized B_1_+ mapping method (Lutti et al., 2010). Using vendor specific protocols for B_1_+ and MT_sat_ mapping may influence the results (Leutritz et al., 2020). Moreover, the calibration factor in Equation (1) may have to be recalibrated for different MT-pulses.

Future studies should investigate the effect of B_1_+ correction on MR g-ratio mapping when using alternative biomarkers to estimate AVF_MR_ and MVF_MR_ (e.g., Ellerbrock and Mohammadi, 2018). Moreover, there are alternative B_1_+ mapping approaches available which might vary in precision (Lutti et al., 2010) and therefore can affect the MR g-ratio values. However, the differences in the precision of these methods are in the order of few percentage and thus much smaller than the effect of omitting the B_1_+ field or using the data-driven UNICORT B_1_+ estimate (Weiskopf et al., 2011).

## Conclusion

In this study, we assessed the effect of B_1_+ correction on the accuracy of MR g-ratio as well as axonal and myelin volume fraction based on MT_sat_ and NODDI. Our results demonstrate that B_1_+ correction via a measured B_1_+ field map is the method of choice. If the B_1_+ field map cannot be acquired, we propose the retrospective, data-driven UNICORT B_1_+ correction to estimate and correct for B_1_+ field inhomogeneities, which reduces the error and bias by a factor of three. UNICORT is implemented in the free and open-source hMRI toolbox (see text footnote 1).

## Data Availability Statement

The datasets presented in this article are not readily available because the data that support the findings of this study are available on request from the corresponding author. The data have not been made freely available on the internet due to privacy or ethical restrictions. Requests to access the datasets should be directed to corresponding author.

## Ethics Statement

The studies involving human participants were reviewed and approved by the Ärztekammer Hamburg. The patients/participants provided their written informed consent to participate in this study.

## Author Contributions

SM and TE contributed to the conception and design of the study, performed statistical analysis and MRI processing, and wrote the first draft of the manuscript. All authors contributed substantially to revising the manuscript critically for intellectual content and have approved the submitted version.

## Conflict of Interest

The authors declare that the research was conducted in the absence of any commercial or financial relationships that could be construed as a potential conflict of interest.

## Footnotes

1
www.hMRI.info

2
http://www.diffusiontools.com
