# Corrigendum: The Influence of Radio-Frequency Transmit Field Inhomogeneities on the Accuracy of G-ratio Weighted Imaging

**DOI:** 10.3389/fnins.2021.772745

**Published:** 2021-10-07

**Authors:** Tim M. Emmenegger, Gergely David, Mohammad Ashtarayeh, Francisco J. Fritz, Isabel Ellerbrock, Gunther Helms, Evelyne Balteau, Patrick Freund, Siawoosh Mohammadi

**Affiliations:** ^1^Spinal Cord Injury Center Balgrist, University Hospital Zurich, University of Zurich, Zurich, Switzerland; ^2^Department of Systems Neuroscience, University Medical Center Hamburg-Eppendorf, Hamburg, Germany; ^3^Department of Clinical Neuroscience, Karolinska Institutet, Stockholm, Sweden; ^4^Medical Radiation Physics, Clinical Sciences Lund (IKVL), Lund University, Lund, Sweden; ^5^GIGA Institute, University of Liège, Liège, Belgium; ^6^Department of Neurophysics, Max Planck Institute for Human Cognitive and Brain Sciences, Leipzig, Germany; ^7^Wellcome Trust Centre for Neuroimaging, University College London, London, United Kingdom

**Keywords:** myelin volume fraction, axon volume fraction, radio-frequency transmit field inhomogeneities, B_1_+ correction, multi-parameter mapping, diffusion MRI, magnetization transfer saturation, MR g-ratio

In the original article, there was a mistake in Figure 5 as published. The MVF map was shown twice in the original figure (middle and bottom rows), instead the AVF map should have been depicted in the middle row. The corrected Figure 5 that appears below.

**Figure 5:**
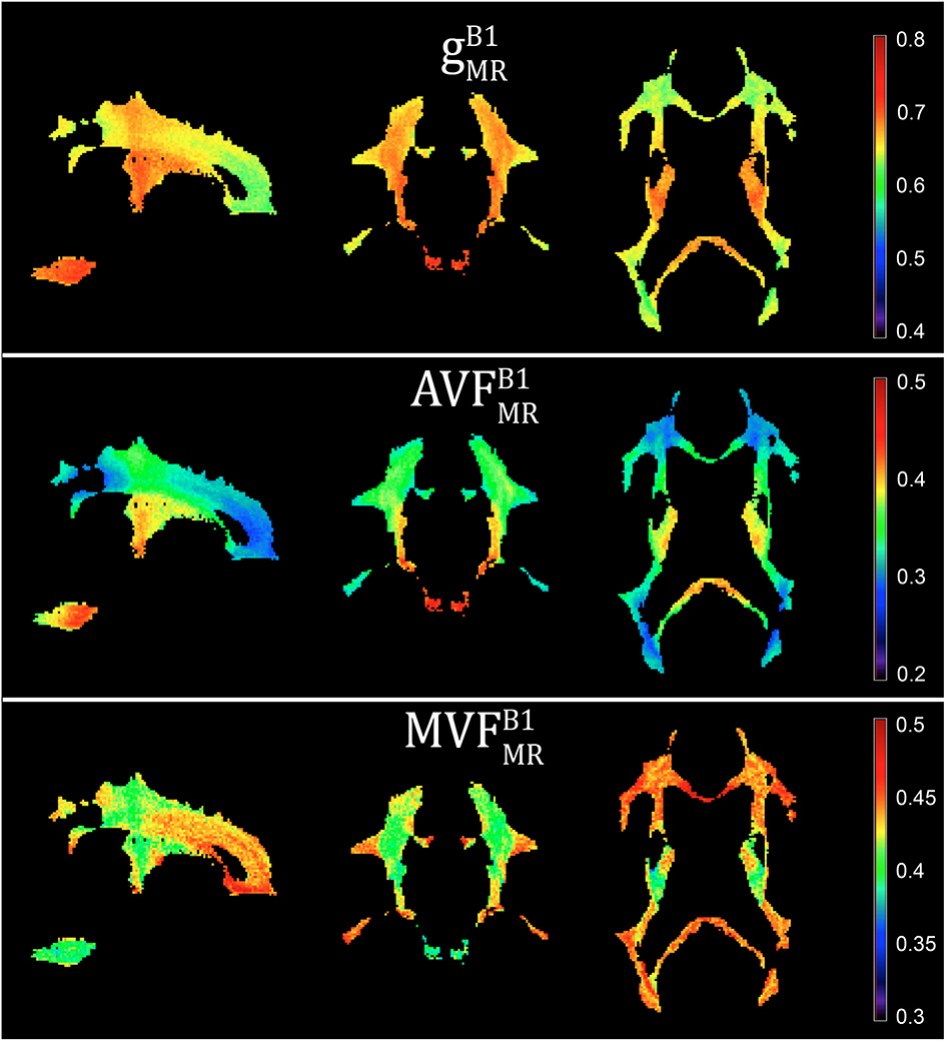
Voxel-wise maps of group-averaged g^B1^_MR_, AVF^B1^_MR_, and MVF^B1^_MR_, restricted to the group WM mask (cf. section “Definition of White Matter Masks”). Depicted are a single sagittal (x = 100), coronal (y = 91), and axial (z = 85) slice.

In the published article, there was an error regarding the affiliation for Isabel Ellerbrock. As well as having affiliation 3, she should also have 2. There was an error regarding the affiliation for Gergely David. As well as having affiliation 1, he should also have 2.

The authors apologize for this error and state that this does not change the scientific conclusions of the article in any way. The original article has been updated.

## Publisher's Note

All claims expressed in this article are solely those of the authors and do not necessarily represent those of their affiliated organizations, or those of the publisher, the editors and the reviewers. Any product that may be evaluated in this article, or claim that may be made by its manufacturer, is not guaranteed or endorsed by the publisher.

