# Supplementary material for: The Influence of Radio-Frequency Transmit Field Inhomogeneities on the Accuracy of G-ratio Weighted Imaging
Source: Front Neurosci. 2021 Jul 5;15:674719. doi: 10.3389/fnins.2021.674719 (PMC8287210; doi:10.3389/fnins.2021.674719)
Supplement: Supplementary file 1 [file Image_1.pdf]

Supplementary Materials

S.1 Whole white matter analysis

Figure S1 summarises the error and bias in $\mathrm{MVF_{MR}}$ when the whole-WM ROIs were used. Across the whole-WM ROIs, the dynamic range of $\mathrm{MVF_{MR}^{B1}}$ was 0.091 with a maximum value of 0.445 in the genu of the corpus callosum and a minimum value of 0.354 in the left median lemniscus, and with mean and standard deviation of 0.411 ± 0.026. With respect to $\mathrm{MVF_{MR}^{B1}}$, the relative error of $\mathrm{MVF_{MR}^{NO}}$ was 52.8% with a positive relative bias of 36.5%, whereas the relative error and bias for $\mathrm{MVF_{MR}^{UN}}$ were smaller ($\epsilon_{\mathrm{DR\%}}$ = 23.96 %;$\bar{\delta}_{\mathrm{DR\%}}$ = -13.08%).

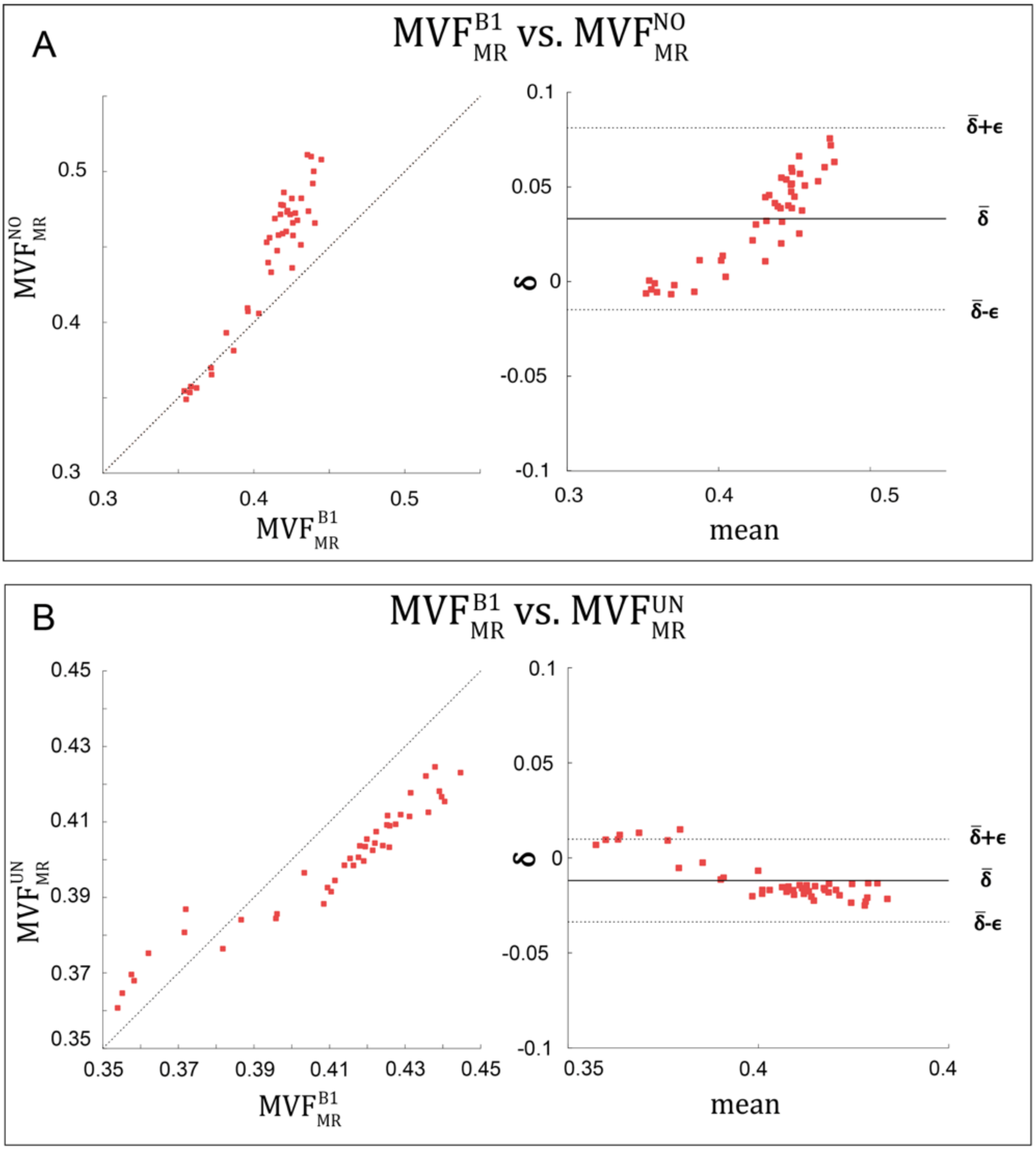

Figure S1 Depicted are scatter (left column) and Bland-Altman (right column) plots of $\mathrm{MVF_{MR}}$ without (superscript: NO, top row) or with UNICORT B_1+ correction (superscript: UN, bottom row) B_1+ field correction against the reference method (superscript: B_1) across 43 WM regions (denoted whole-WM ROIs). The Bland-Altman plot illustrates the differences between values obtained by two different methods (reference vs. tested method; e.g., $\delta_i = (\mathrm{g_{MR}^{B1}})_i - (\mathrm{g_{MR}^{k}})_i$ against their mean (e.g. $\mathrm{mean}_i = \frac{(\mathrm{g_{MR}^{B1}})_i + (\mathrm{g_{MR}^{k}})_i}{2}$, with $\mathrm{k} = \{\mathrm{UN}, \mathrm{NO}\}$ and i indexing the ith ROI). Each point in the scatter plot represents the group-averaged value in a single ROI (see Figure 4 for the locations of the 21 high-SNR ROIs). The bold black line represents the bias ($\bar{\delta} = \sum_{i=1}^{21} \delta_i$), while the dashed line shows error ($\epsilon = \mathrm{SD}(\delta_i)$) between the reference and the tested method. Error and bias values averaged across all ROIs and subjects are listed in Table 1. The errors and biases are summarised in Table 4.

S.2 How to apply UNICORT correction on MT_{sat}

MT_{sat} maps are generated using the hMRI toolbox. To enable UNICORT B_1+ correction, the hMRI toolbox must be configured (https://github.com/hMRI-group/hMRI-toolbox/wiki/DefaultsAndCustomization) (Figure S2A). The following steps assume you have installed the hMRI toolbox (https://github.com/hMRI-group/hMRI-toolbox/wiki/GetStarted) and opened the SPM Batch GUI in Matlab (Figure S2B). The “Configure toolbox” module must be run prior to the “Create hMRI maps” module. In “Configure toolbox”, select “Defaults parameters > Customised”, then upload the customised defaults file (Figure S2C). The customised defaults file must enable the following parameter: “UNICORT.MT = true;”. Such a default file is available as supplementary materials S_default. In “Create hMRI maps” (Figure S2D), under “B1 bias correction”, “UNICORT” must be selected (Figure S2E). An example batch file for a single subject is also available as S_batch. Be aware that these example defaults and batch files may need to be further customised to match your own data.

Note that it’s not good practice to modify this local defaults file. Instead it should be saved under another meaningful name, and stripped from all the lines that are not modified (as done in this example), so one can directly see what is customised. Type “help hmri_defaults” in Matlab to be led to the full explanation (header of hmri_local_defaults.m).

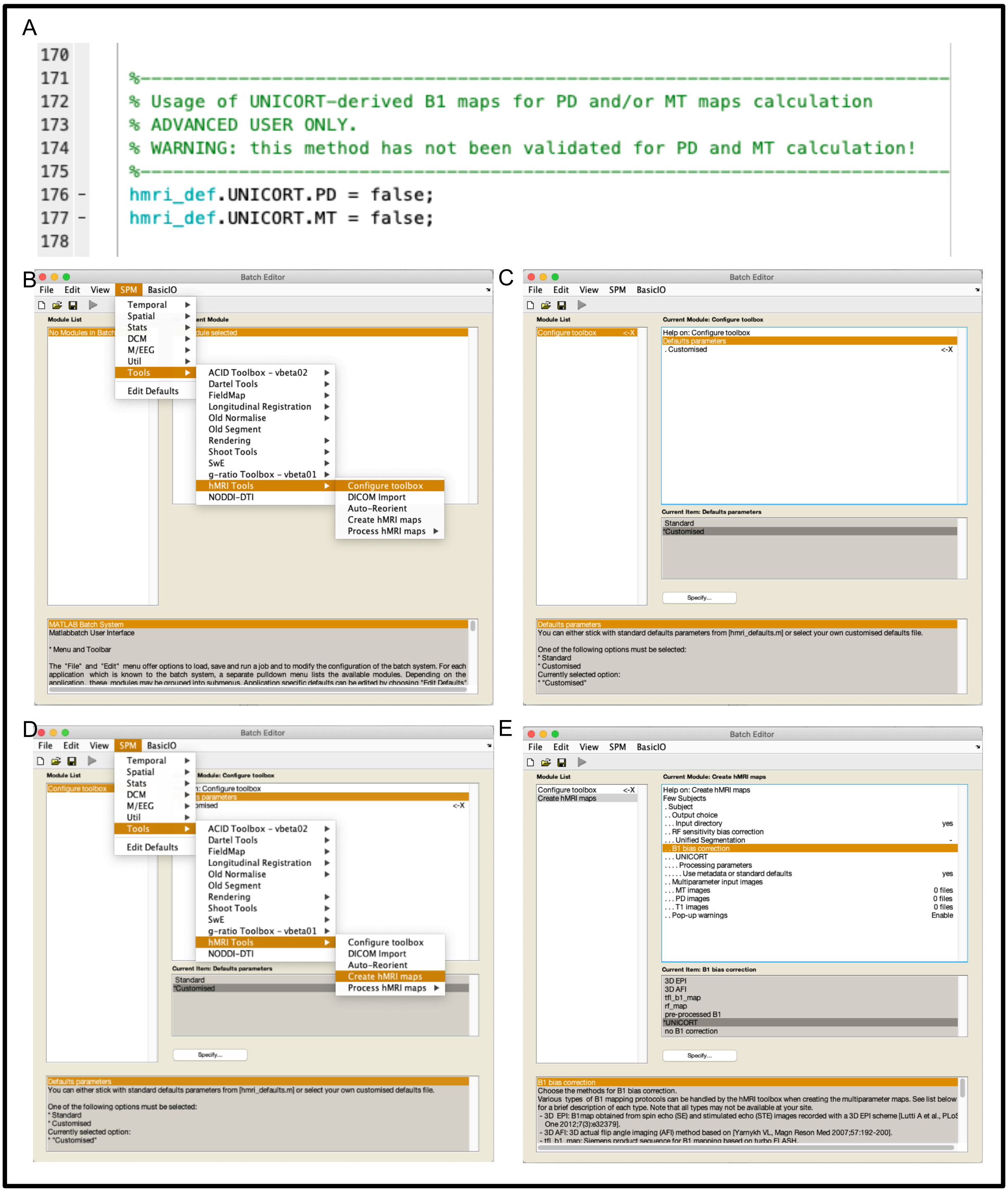

Figure S2 Necessary steps to enable the UNICORT correction on MT_{sat} images. (A) To enable UNICORT correction, the depicted line has to be changed from false to true in the hmri_local_defaults.m file in your spm12/toolbox/hMRI/config/local directory or the customized file – see S_defaults used. (B) To use the updated hMRI settings, the configuration toolbox function has to be opened. (C) In this function, the defaults parameters have to be changed from standard to Customised. For the Customised settings the modified hmri_local_defaults.m file (or S_defaults) used and the modified hmri_local_defaults.m has to be chosen. (D) To generate the MT_{sat} images the Create hMRI maps function has to be opened, and for the B_1+ bias correction, UNICORT correction has to be chosen.
